# Nanostructured Silica/Gold-Cellulose-Bonded Amino-POSS Hybrid Composite via Sol-Gel Process and Its Properties

**DOI:** 10.1186/s11671-017-2122-9

**Published:** 2017-06-02

**Authors:** Sivalingam Ramesh, Heung Soo Kim, Young-Jun Lee, Gwang-Wook Hong, Joo-Hyung Kim

**Affiliations:** 1 Department of Mechanical, Robotics and Energy Engineering, Dongguk University-Seoul, Pil-dong, Jung-gu, Seoul, 100-715 South Korea; 2 Department of Mechanical Engineering, Inha University, Inha-ro 100, Nam-gu, Incheon, 402-751 South Korea

**Keywords:** Cellulose, POSS, Silica/gold, Core/shell nanoparticles, Optical transparency

## Abstract

It is demonstrated in this paper that silica nanoparticles coated with core/shell gold provide efficient thermal, optical, and morphological properties with respect to the cellulose-polyhedral oligomeric silsesquioxanes (POSS) hybrid system. The one-step synthesis of a silica/gold nanocomposite is achieved with a simultaneous hydrolysis and reduction of gold chloride in the presence of formic acid, and the trimethoxysilane group acts as a silica precursor. The focus here comprises the synthesis of cellulose-POSS and silica/gold hybrid nanocomposites using the following two methods: (1) an in situ sol-gel process and (2) a polyvinyl alcohol/tetrakis (hydroxymethyl)phosphonium chloride process. Accordingly, the silica/gold core/shell nanoparticles are synthesized. The growth and attachment of the gold nanoparticles onto the functionalized surface of the silica at the nanometer scale is achieved via both the sol-gel and the tetrakis (hydroxymethyl) phosphonium chloride processes. The cellulose-POSS-silica/gold nanocomposites are characterized according to Fourier transformed infrared spectroscopy, Raman, X-ray diffraction, UV, photoluminescence, SEM, energy-dispersive X-ray spectroscopy, TEM, thermogravimetric, and Brunauer-Emmett-Teller analyses.

## Background

The field of nanotechnology is one of the most popular current-research areas, and it is being developed in chemistry, physics, biology, and materials science; here, polymer science and technology are obviously included, as well as a broad range of topics. This area of research was utilized for microelectronics and nanoelectronics, as the critical dimension scale for modern devices is now less than 100 nm [1, 2]. Therefore, the synthesis protocols of metal-oxide-hybrid composites are already well established in the literature [2, 3] and most of them are multistep metal-nanoparticle processes. The synthesis of the silica/gold hybrid composite materials is achieved using the in situ *sol-gel process* via the hydrolysis of gold and silica precursors into a cellulose-polyhedral oligomeric silsesquioxanes (POSS) matrix [3–5].

The gold nanoparticles have recently been synthesized by the reduction of chloroaurate (HAuCl_4_) ions for which different methods such as those involving sodium borohydrate, citrate, and other reducing agents are used [6, 7]. Based on this synthesis process, the stabilizing agents such as thiols, amines, phosphines, phosphine oxides, and carboxylates have been used to control the morphology of the nanoparticles. In addition, cellulose constitutes the most abundant, currently available renewable-polymer resource material, and it has received great attention due to its renewability, availability, non-toxicity, low cost, biodegradability, thermal stability, and chemical stability [8, 9]. Moreover, the polyhedral oligomeric silsesquioxanes (POSS) comprise nanostructures that contain the empirical formula RSiO_1.5_, where R may be a hydrogen atom or an organic functional group, e.g., alkyl, alkylene, acrylate, and hydroxyl functional groups [10, 11]. The focus of the cellulose-metal-oxide hybrid is the synthesis of the uniform dispersion of nanoparticles in the composite that is utilized for flexible electronic devices, chemical sensors, disposable sensors, and biosensors [12–14]. The sol-gel chemistry to synthesize the cellulose-binary mixed oxides has been widely reported in the literature. The focus of the present study is the synthesis of cellulose-POSS silica/gold that is covalently bonded by an in situ sol-gel process that includes the involvement of surface-modified PVA and tetrakis (hydroxymethyl) phosphonium chloride (THPC) in the hybrid composites. Based on the two chemical processes in the presence of tetra ethoxysilane (TEOS), chlorauric acid (HAuCl_4_) and γ-aminopropyl triethoxysilane (γ-APTES) are bonded to the cellulose-POSS hybrid nanocomposites. The cellulose-POSS-silica/gold hybrid nanocomposites are characterized by Fourier transformed infrared spectroscopy (FT-IR), X-ray diffraction (XRD), Ultraviolet-visible spectral (UV-VIS), scanning electron microscopy-energy-dispersive X-ray spectroscopy (SEM-EDX), SEM, Brunauer-Emmett-Teller (BET), and transmission electron microscopy (TEM) analyses.

## Methods

### Materials and Methods

The cotton cellulose with a specific degree of polymerization (DP = 4500) was purchased from Buckeye Technologies Co., (USA). Lithium chloride was purchased from Junsei Chemical Japan. The cotton pulp (buckeye) is purified in the presence of the LiCl, and sulfuric acid is used in the synthesis of the cellulose solution. The molecular sieves (containing 4A°, four meshes to eight meshes) that are used for the additional purification were received from Acros Organics Ltd, New Jersey, USA. Dimethylacetamide (DMAc) (anhydrous, 99.8%) was received from Sigma-Aldrich, USA. The cotton pulp was mixed with LiCl/anhydrous DMAc according to a proportion of the cotton-cellulose pulp/LiCl/DMAc that is 2/8/90 by mass. The cotton pulp and LiCl in the presence of sulfuric acid was used to purify the cellulose solution from the bulk-cotton fibers. The cellulose solution, PSS [3-(2- amino ethyl) amino] propyl-Hepta isobutyl substituted (POSS-amine), tetra ethoxysilane (TEOS), chlorauric acid (HAuCl_4_), γ-aminopropyl triethoxysilane (γ-APTES), hydrocholoric acid (HCl), poly (vinyl alcohol) (PVA), and tetrakis (hydroxymethyl) phosphonium chloride (THPC) were purchased from Aldrich (South Korea).

### Synthesis of Cellulose-POSS-Amine-Silica/Gold Hybrid Nanocomposites

**Fig. 1:**
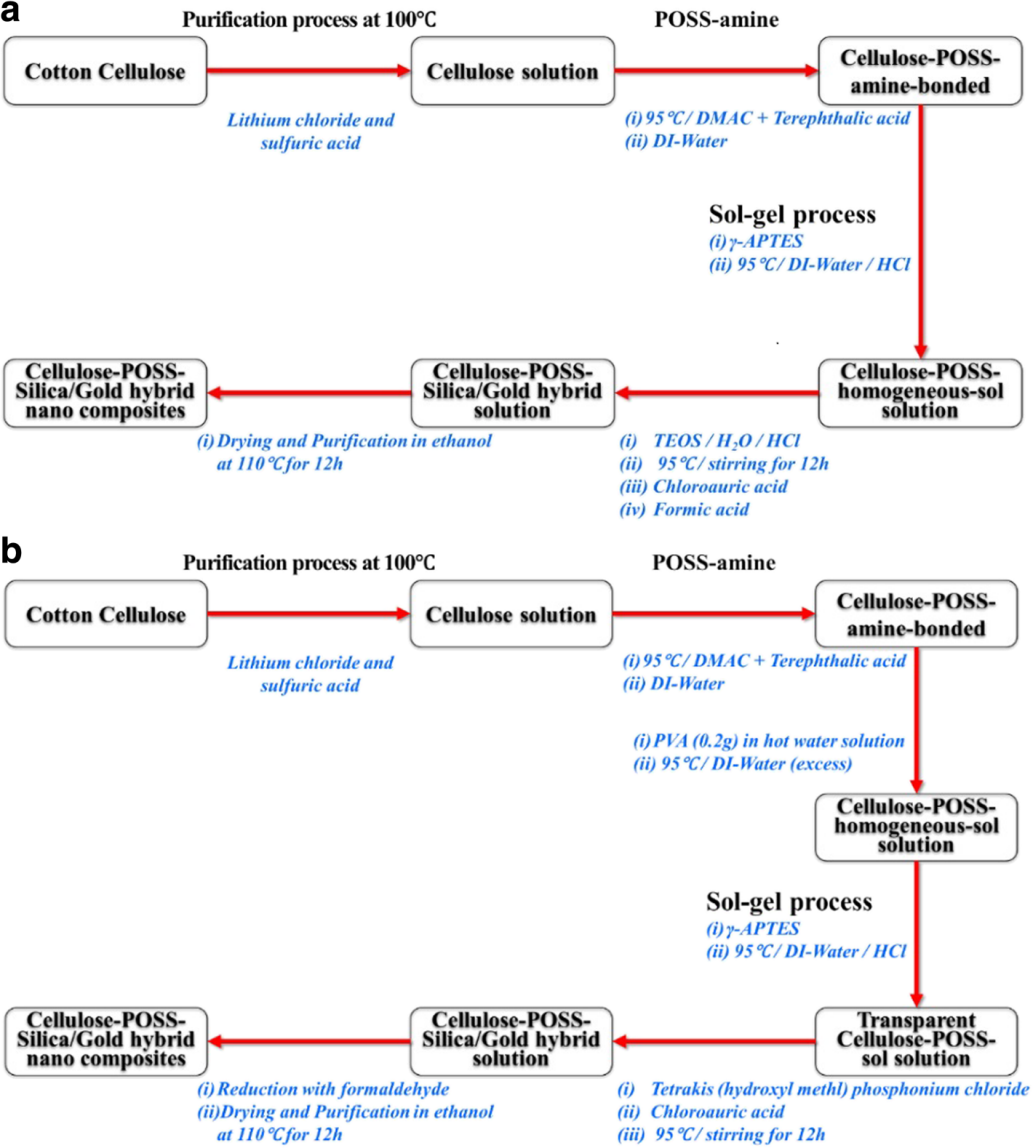
Synthesis of cellulose-amino POSS hybrid composite by (a) sol-gel process (b) PVA/THPC process

The cellulose-POSS-silica/gold hybrid nanocomposites are synthesized using two chemical methods (Fig. 1a, b) as follows: *Method 1.* The stoichiometric amount of the cellulose solution (0.5 g) and the POSS-amine (0.35 g) are dissolved in DMAc (50 ml) and stirred (300 rpm) for 1 h in the presence of terphthalic acid (0.5 g), followed by the continuous stirring of the mixture for another 2 h at 95 °C until the homogeneous solution is achieved. The reaction is followed by an application of the same temperature, and the calculated γ-APTES amount of (2 ml) is mixed and stirred for 2 h to obtain the homogenous solution. Then, the calculated amount of TEOS (2 g) and an equal amount of HAuCl_4_ (2 ml, 0.002 mM, and 0.004 mM) are added, followed by the addition of formic acid and distilled water (10 g), and they are mixed and continuously stirred at 95 °C for 12 h. The resulting solution is of a yellow transparent color, but it then changes into a light-pink color, and the reaction mixture is transferred into a beaker and purified in ethanol. The purified product is kept in the oven at 95 °C for 12 h, where the solvent is allowed to evaporate, and it is again purified in ethanol several times. The final product is the cellulose-POSS-silica/gold hybrid nanocomposites.

*Method 2.* The stoichiometric amount of the cellulose solution (0.5 g) and the POSS-amine (0.35 g) are dissolved in (50 ml) of DMAc and then stirred for 1 h in the presence of terphthalic acid (0.5 g). The mixture is then continuously stirred (300 rpm) for another 2 h at 95 °C until the homogeneous solution is achieved. The reaction is followed by an application of the same temperature, and the calculated amount of 0.2 g of PVA in the presence of a hot-water solution is transferred into the reaction mixture to obtain the homogenous solution. The calculated amount (2 ml) of γ-APTES is added into the homogenous-sol-reaction mixture and then dispersed in the same temperature, followed by stirring for 2 h. The required 2 ml of TEOS and 5 ml THPC solution is added along with 2 ml of HAuCl_4_ (2 ml, 0.004 mM), and this is followed by the reduction of the formic acid 5 ml and stirring for 12 h. Further, the reaction mixture is transferred into a beaker, purified in ethanol, and kept in the oven at 95 °C for 12 h. Lastly, the resultant cellulose-POSS-silica/gold hybrid nanocomposites are collected in a sol-gel bottle to avoid the moisture content before the characterization process.

### Measurements and Characterization

#### Fourier transformed infrared spectroscopy (FT-IR) analysis

FT-IR spectra of the cellulose-POSS-silica/gold hybrid composite were recorded by using the Brucker, IF5-859 spectrometer from Digilab (Cambridge, USA) with KBr beam splitter and detector at 8 cm^−1^ resolution.

#### Raman spectral analysis (Raman)

Raman spectral analysis was performed by using the RM200 confocal Raman spectromicroscope scanning from 100 to 400 cm^−1^ at room temperature in open air, and an He-Ne laser beam with a wavelength of 580–600 nm.

#### X-ray diffraction (XRD) analysis

Wide angle XRD pattern of the hybrid composite were recorded with the Riguku co D/max X-ray diffractometer for which Cu Kα radiation. The tube current and the voltage 300 mA and 40 kV, respectively, and data from the 2*θ* angular regions between 5 and 80 °C.

#### Ultraviolet-visible spectral (UV-VIS) and photoluminesence spectral analyses

A UV-VIS spectrophotometer UV6000 was used to analyze absorption spectra of hybrid composite samples. Photoluminescence (PL) spectral results were conducted at room temperature by using a SPEC-1403 PL spectrometer (HORIBA Ltd., Tokyo, Japan) with a He-Cd laser (325 nm) as the excitation source. The power of the He-Cd laser was used 55 mW, and the diameter of the focal spot was 1 mm. The power density at the surface of the sample was approximately 7 W/cm^2^ .

#### Scanning electron microscopy (SEM and EDX)

The collected hybrid composites were characterized by SEM (Hitachi S-4200, Hitachi Ltd., Tokyo, Japan), and the EDX analysis is performed using the AN-ISIS 310.

#### Transmission electron microscopy (TEM)

The transmission electron microscopy of hybrid composite results were obtained using the 100CX electron microscope (JEOL, Ltd., Japan).

#### Thermal properties (thermogravimetric analysis (TGA) and differential scanning calorimetry (DSC))

TGA were carried out using the TA Instruments 2050 Universal V4.1D. Ceramic hybrid samples weighing 9.7 mg are heated up to 1000 °C at 10 °C/min. The DSC analysis of hybrid composite was also carried out using the TA Instruments 2050 Universal V4.1D.

#### Brunauer-Emmett-Teller (BET) analysis

The specific surface area and average pore volume of the hybrid nanocomposites were calculated according to the Brunauer-Emmett-Teller (BET) analysis (BELmax 00131 equipment, BELSORP, Tokyo, Japan).

## Results and Discussion

### Formation of Cellulose-POSS-Silica/Gold Nanoparticle Composite

The macromolecular structure of cellulose is presented with a number of hydroxyl groups, and the POSS-amine can be grafted into the cellulose macromolecular structure in the presence of terphthalic acid. As a crosslinking agent, the acid compound is able to form bonds between the cellulose and the POSS-NH_2_ hybrid compounds. The schematic representations of the cellulose and the POSS-NH_2_ are shown in Fig. 1a, b, respectively. In the graft reaction, the POSS particles are dispersed in the cellulose-host matrix and bond to the cellulose molecule, thereby forming cellulose-POSS hybrids. In addition, the bonding of the silica/gold nanoparticles via the sol-gel process is as follows. The original synthesis of the silica/gold nanoparticles is a four-step process in which the monodisperse-silica nanoparticles are first grown using the Stöber method to produce the spherical dielectric cores of the nanoparticles [13]. The Stöber method produces spherical silica nanoparticles by means of the hydrolysis of alkyl silicates and the subsequent polycondensation of silicic acid in an acid or a base catalyst. In the second step, the surfaces of the silica nanoparticles are functionalized by the adsorption of γ-APTES with its amine tails protruding from the surface of nanoparticles. In the third step, the gold-colloid solution is added to the resultant silica solution. According to the phonthammachai and Jun-hyun Kim reports, the gold colloid is produced separately from the reduction of HAuCl_4_ by the formic acid and alkaline THPC [13–15]. The gold nanoparticles are bonded via the organo-aminosilane groups that produce the silica-hybrid nanoparticle composites. A final reduction process is used to produce silica nanoparticles with a uniform layer of gold nanoshell in the presence of formic acid. In the reduction process, the formed silica/gold particles that are covalently bonded to the silica core serve as nucleation sites for an aged mixture of the chloroauric acid and the reducing agents. *Method 1*. FT-IR spectroscopy is employed to study the chemical structure of the amino-POSS-bonded cellulose-hybrid nanocomposites in the presence of terphthalic acid during the sol-gel process.

**Fig. 2:**
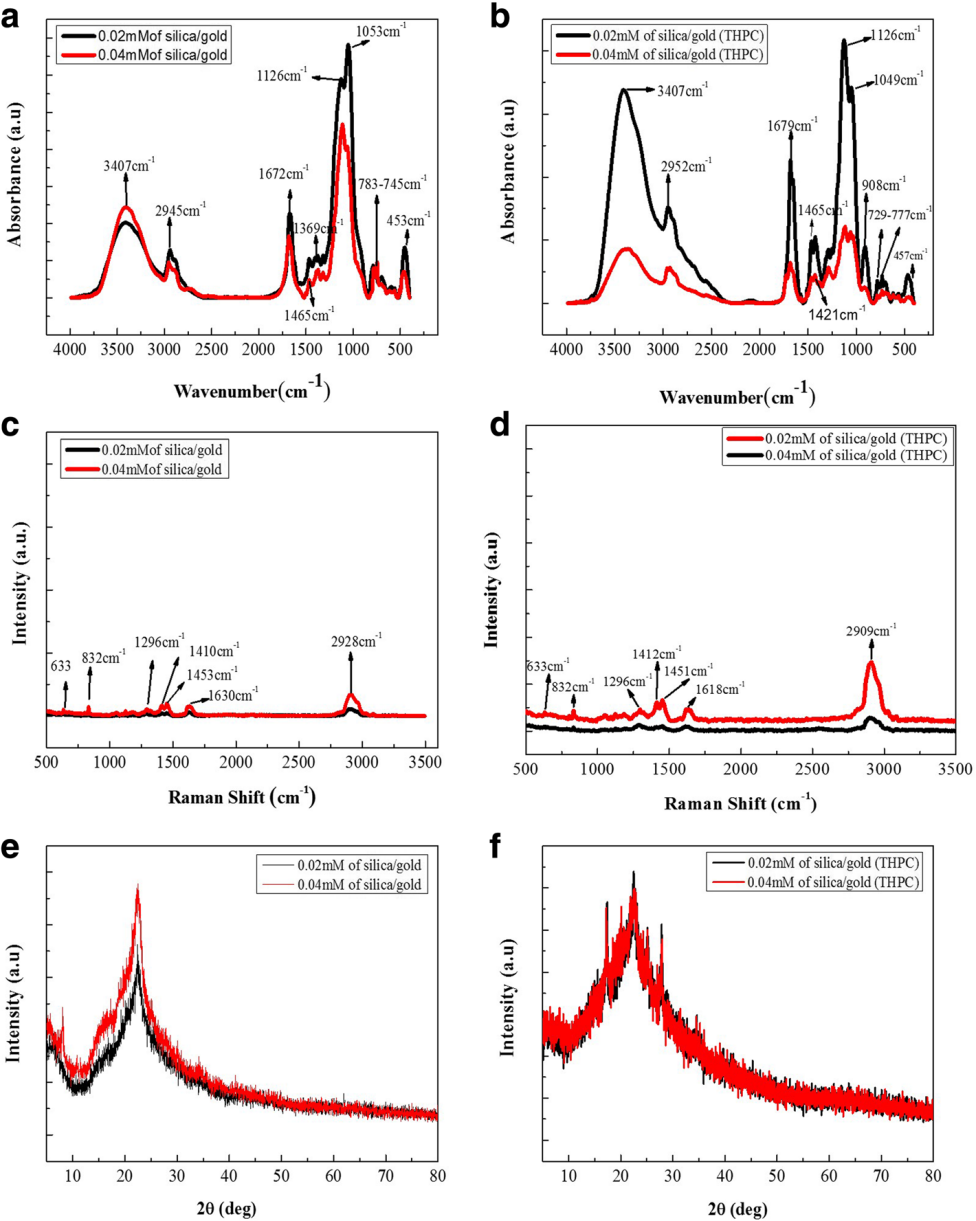
(a-b) FTIR (c-d) Raman (e-f) XRD results of cellulose-amino POSS hybrid composite

In terms of the sol-gel process, the FT-IR spectra (Fig. 2a) of the cellulose hybrids show the bands at the 3407 cm^−1^ (N–H) bonded and unreacted OH groups from the cellulose, and the 2945 cm^−1^ (CH,CH_2_ groups), 1672 cm^−1^ (C=O,CO), 1369 cm^−1^ (CO–NH), 1465 cm^−1^ (phenyl group from terphthalic acid), 1126 cm^−1^ (Si–O–Si,Si–O–Au), 1053 cm^−1^ (Si–O–C), 783–745 cm^−1^ (C–H bending), and 453 cm^−1^ (Au–O starching) frequencies of the cellulose-POSS-bonded silica/gold hybrid nanocomposites are shown in Fig. 2b. *Method 2*. The FT-IR spectral analysis (Fig. 2b) and the Raman spectral values (Fig. 2c, d) of the cellulose-POSS-silica/gold hybrid nanocomposites are shown in the presence of PVA and THPC. In this method, the cellulose hybrids exhibit that the bands at the 3407 cm^−1^ (N–H) bonded and unreacted (OH) groups from the cellulose, and the 2952 cm^−1^ (CH,CH_2_ groups), 1679 cm^−1^ (C=O,CO), 1369 cm^−1^ (CO–NH), 1421–1465 cm^−1^ (phenyl group from terphthalic acid), 1126 cm^−1^ (Si–O–Si,Si–O–Au), 1049 cm^−1^ (Si–O–C), 777–729 cm^−1^ (C–H bending), and 457 cm^−1^ (Au–O starching) frequencies are almost similar to the bonding behavior of the cellulose-POSS-bonded silica/gold hybrid nanocomposites.

### X-ray Diffraction (XRD) Analysis

The XRD study for which the cellulose-POSS-silica/gold hybrid composites are synthesized by the sol-gel process is shown in Fig. 2e. The results indicate the values of 2*θ* = 22.56°, 25.14°, 27.90°, 30.08° (less intensity) for the silica/gold formation, and the board peaks of 8°, 17°, and 21° for the regeneration of the cellulose-POSS matrix. The XRD peak values indicate the planes of the face-centered cubic (fcc) gold structure (JCPDS 04- 0784), which indicate the crystalline behavior of the silica/gold nano-hybrid core/shell growth in the nanoparticle composite. The silica/gold core/shell hybrid nanocomposites show two phases, namely the fcc of Au and the tetragonal. This shows that the coated gold nanoparticles induce the silica crystallization at a lower temperature in situ during the sol-gel process and control the morphology [13–15]. Therefore, the metal disrupts the amorphous network, reducing the kinetic barrier regarding the crystallization. *Method 2.* Figure 2f shows the XRD study of the cellulose-POSS-silica/gold hybrid materials that are synthesized using PVA and THPC. The regeneration-peak value of the cellulose-POSS is 2*θ* = 7.96°. The other peak values of 2*θ* = 17.34°, 22.54°, 25.12°, and 27.88° (sharp peak) (silica/gold formation) represent the crystalline behaviors of the hybrid nanocomposites.

### UV-Visible Spectral (UV-VIS) Analysis

**Fig. 3:**
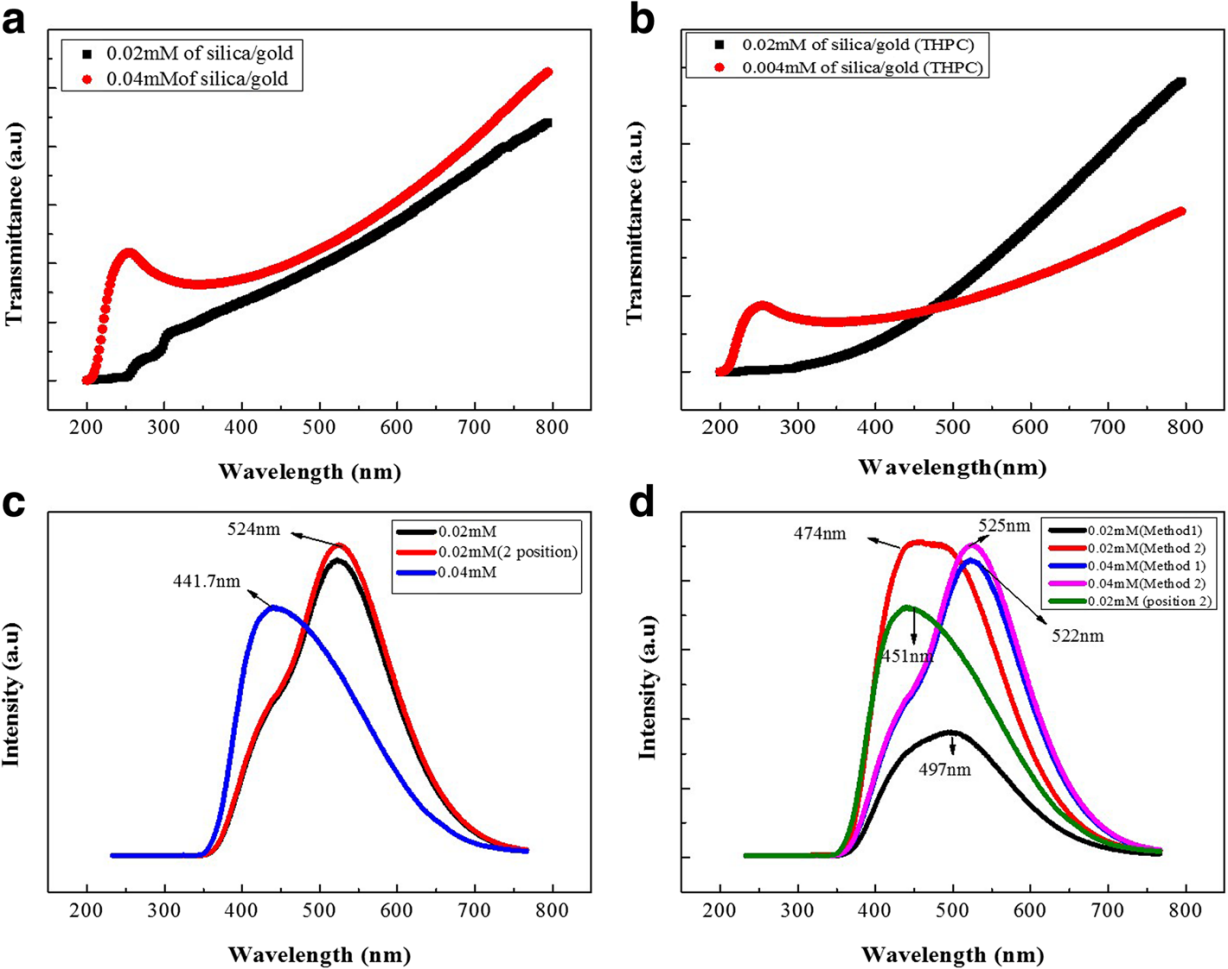
(a-b) UV (c-d) PL spectral results of cellulose-amino POSS hybrid composite

The cellulose-POSS-silica/gold hybrid nanocomposites are synthesized by two methods as follows: (1) in situ sol-gel process and (2) surface-modifying PVA-THPC method. From this chemical-modification protocol that is in accordance with a different temperature process, the formation of the hybrid nanocomposites are studied regarding turbid, transparent, and translucent behaviors in the UV spectral analysis. The transparent properties of the hybrid nanocomposites in terms of the optical applications show that the high optical of the silica/gold hybrid nanocomposites is due to the transparency and the surface-modified properties that are occurred during the sol-gel process. The UV-transmittance results for the cellulose-POSS-silica/gold hybrid nanocomposites regarding the in situ sol-gel process are shown in Fig. 3a, b. The formations of the hybrid composites with and without coupling agents are used to characterize the surface of the cellulose-POSS silica via a crosslinking process. The hybrid composite shows the core/shell nanoparticles in the uniform composite morphology that are due to alkoxysilanes and are highly dependent on the temperature, whereby a faster gelation and a greater particle size are observed as the temperature increases. At low temperatures from 50 to 70 °C, a longer gelation rate of the TEOS is observed due to a declining of the homogeneity to l, and the gold precursors of the hybrid nanocomposites are observed during the hydrolysis process due to the growing nanoparticles; however, the agglomeration disappears along with the process-temperature increase, whereby the hybrid sol solution becomes homogeneous or transparent between 90 and 95 °C. The results indicate that kinetic control can play an important role in the formation of the optical transparency of the hybrid nanocomposites during the chemical process. The hybrid formation is originally immiscible, phase-separated, or transparent depending on the temperature and the control of the pH in the reaction of the sol-gel process. The synthesis of the cellulose-POSS-silica/gold hybrid is heated between 90 and 95 °C in the reaction mixture with different molar concentrations via the sol-gel process in the presence of an acid catalyst. The degree of hydrolysis increases again with the increasing of the amounts of the acid catalyst and the hybrid nanocomposites, thereby controlling the high uniformity without any phase separation. The silica coating of the gold nanoparticles is accomplished using the classic Stöber method, followed by the application of tetraethyl orthosilicate (TEOS), whereby a highly branched and mesoporous siloxane polymer is formed on the surface of the gold. The reaction can be controlled so that the thickness of the silica layer on the gold surface can be tailored according to the reaction parameters [6, 7, 13–15]

### Photoluminescence (PL) Properties

Figure 3c, d shows the PL spectra of the cellulose-POSS-silica/gold hybrid nanocomposites according to the sol-gel process. In this process, different amounts of the silica and gold nanoparticles (0.002 and 0.004 M, respectively) are present in the hybrid nanocomposites. The PL spectra results show the sharp peak in the red-band region of the fundamental absorption, and the peaks centered at 441.7, 451, 474, and 497 nm indicate the silica-based electrons. Another emission from the PL spectra shows the band gap between 2.3 and 2.80 eV (524 nm) wherein the broad and intense peaks of gold nanoparticles of different sizes are present. The smaller size indicates that the origin of these bands comes from the exciting laser and is penetrated through the porous layer of the gold nanoparticles, and the optical-coupling properties have been discussed in detail in previous studies [13–15]. The gold-crystallite size therefore becomes smaller, and the intensity in the PL properties becomes higher and stronger. It is also noted that the plasmon peak exhibits a blue shift with the decreasing of the particle size. The observed blue shift in the peak position of the plasmon absorption is due to the quantum-size effects from the gold nanoparticles.

### SEM, SEM-EDX, and TEM Analyses

**Fig. 4:**
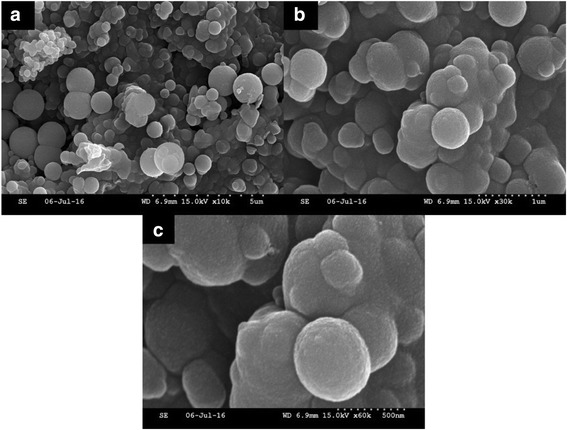
(a-c) SEM morphology of cellulose-amino POSS hybrid composite

**Fig. 5:**
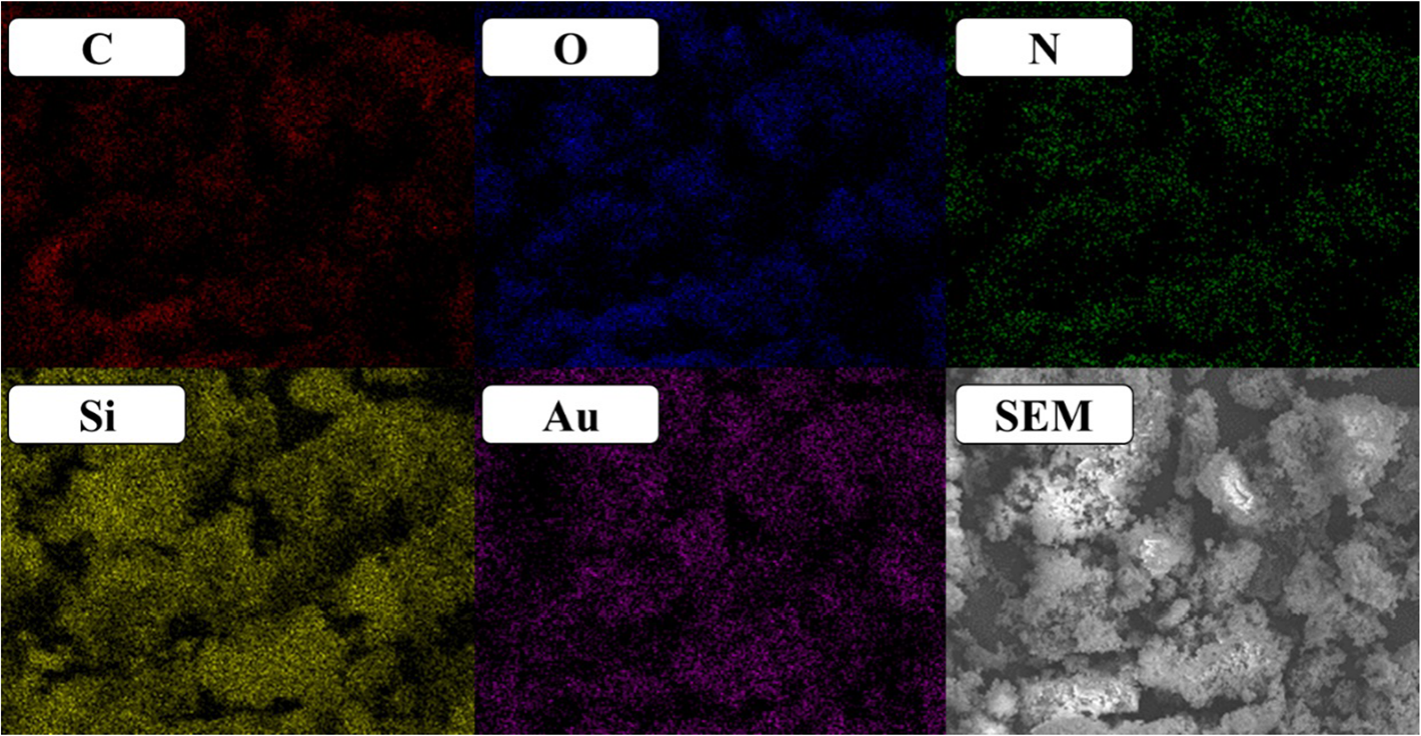
SEM-EDX mapping results of cellulose-amino POSS hybrid composite by sol-gel process

**Fig. 6:**
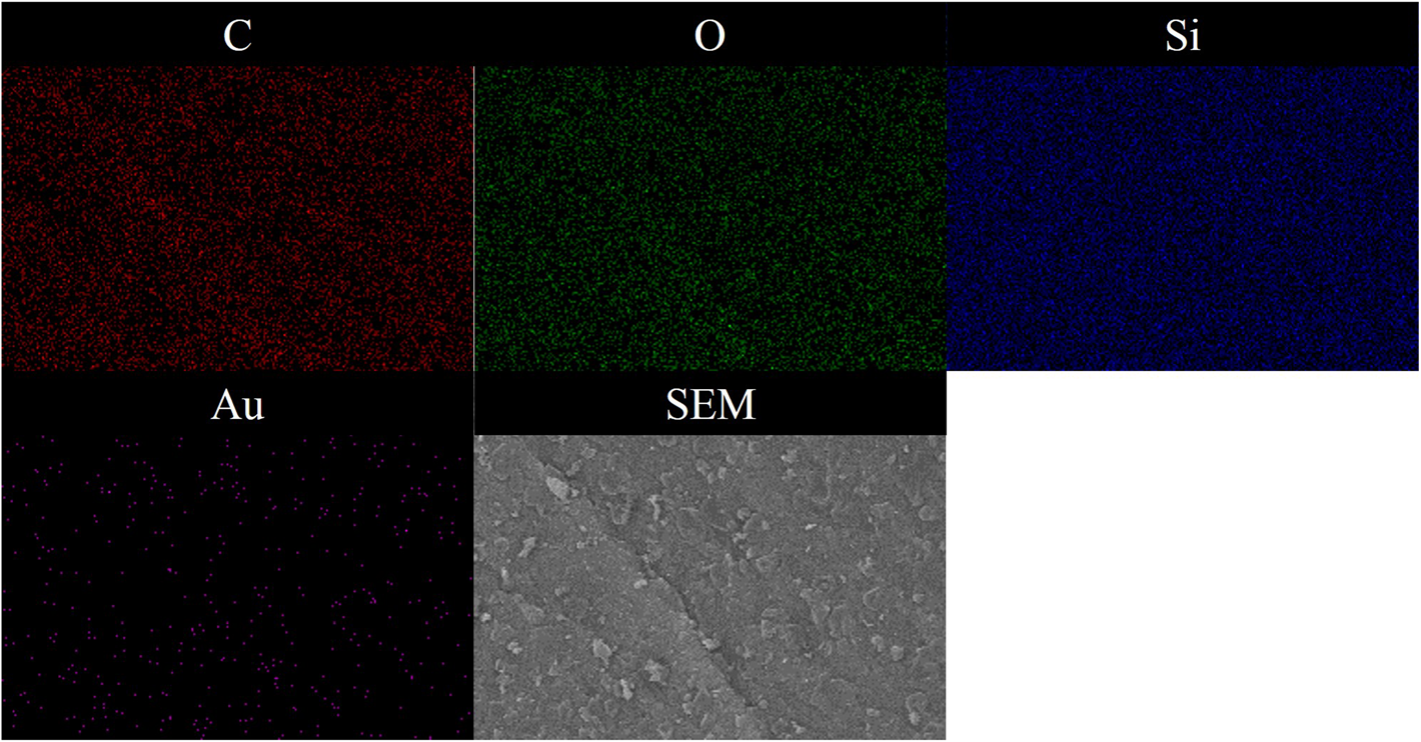
SEM-EDX mapping results of cellulose-amino POSS hybrid composite by PVA/THPC process

The SEM, SEM-EDX, and TEM (Figs. 4, 5, and 6) results show the surface morphology of the cellulose-POSS-silica/gold hybrid nanocomposites that are achieved via the covalent-crosslinking sol-gel process. Figures 4, 5, and 6 show the SEM and SEM-EDX micrographs that are taken at different magnifications of the hybrids. The SEM results indicate that the different magnifications of the cellulose-POSS-silica/gold hybrid nanocomposites with the coupling agent show that the hybrid nanocomposites are transparent, and the controlled particle size is due to the formation of the core/shell silica/gold nanoparticles. Meanwhile, for the case without the coupling agent, the particle agglomeration and the formation of the hybrid nanocomposites are of greater sizes and show heterogeneous structures. The monodispersed Au-SiO_2_ colloids are successfully prepared via a direct Stöber synthetic procedure that is followed by the sol-gel method (0.02 and 0.04 mM). For this method, a silica-shell thickness in the range of tens to hundreds of nanometers in the presence of coupling agents and the TEOS and gold-nanoparticle concentrations are reported elsewhere [6, 7, 13–15].

**Fig. 7:**
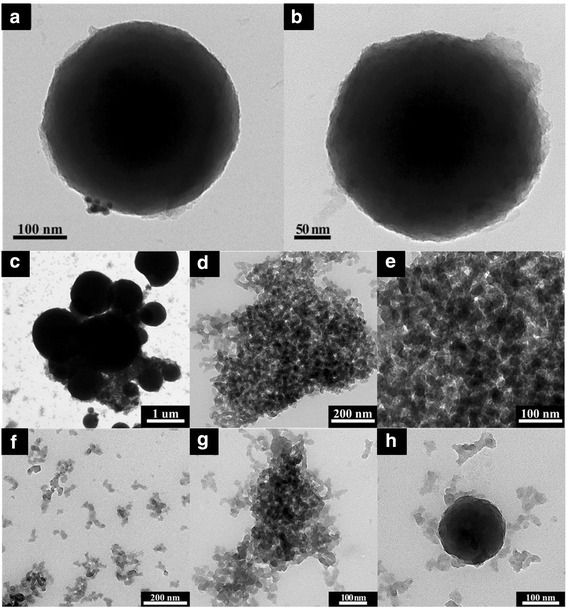
TEM morphology of cellulose-amino POSS hybrid composite (a-c) sol-gel process (d-h) PVA/THPC process

**Fig. 8:**
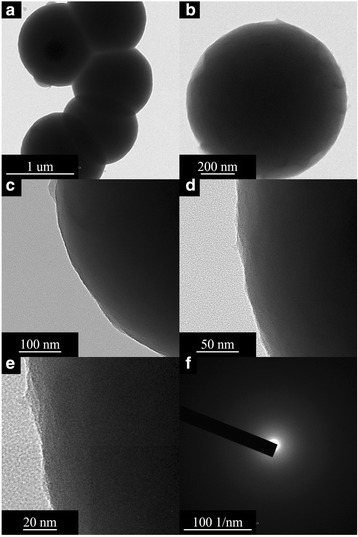
FE-TEM morphology of cellulose-amino POSS hybrid composite (a-e) sol-gel process (f) SAED pattern

**Fig. 9:**
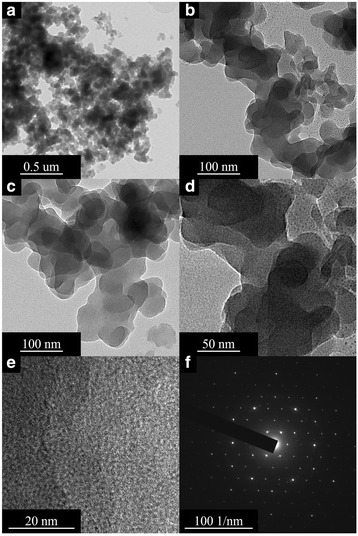
FE-TEM morphology of cellulose-amino POSS hybrid composite (a-e) PVA/THPC (f) SAED pattern

The TEM (Fig. 7a–f) results are recorded prior to the coating in the presence of the Au-SiO_2_ colloids with different silica-shell thicknesses, the diameter of the Au core is 50 nm based on the TEM results that are in accordance with the TEOS concentration, and the thickness of the silica shell could be varied from 20 to 100 nm. From the HR-TEM (Figs. 8a–f and 9a–f) results, an observation that is different from the results of the previous related studies on the hybrid silica nanoparticle in composites becomes evident. In the first stage, only a part of the gold-nanoparticle surfaces seems to be covered by the amorphous silica of a 10–20-nm thickness through the use of higher TEOS concentrations, and a more-complete silica shell for which the thickness is from 20 to 40 nm is formed. Moreover, the core/shell colloids exhibited the original shape of the Au cores, and a relatively large variation of the size in the hybrid samples. Lastly, when the TEOS concentration is further increased, the silica shell grows thicker to a size between 50 and 100 nm, and a more-uniform and smooth surface is observed [5–7, 13–15]. The TEM micrograph (Fig. 7f–h) shows that the range of the 500-nm uncoated silica sphere is smooth and monodispersed prior to the gold-nanoparticle coating in the presence of surface modifiers, which is for the controlling of the morphology of the gold in the 2–4 nm. This may be because the lowest particle size of the nanoparticles in the silica/gold hybrid composite is observed in the presence of strong cohesive interactions that are between the organic/inorganic particles via the coupling agent. The cellulose-POSS-silica/gold hybrid nanocomposites are therefore homogeneously dispersed in the nanoparticles and the formation of the hybrid nanostructure. This may be molecular-level dispersion through the surface morphology by the sol-gel process, and the surface-modified silica/gold in the presence of PVA and THPC. In addition to the gold nanoparticles on the surface of the aminopropyl-modified silica particles that are discussed in the experimental section, the colloidal solution of gold was used to deposit Au nanoparticles (5–10 nm) on the aminopropyl-modified surfaces of the 500-nm silica nanoparticles. This reduction process leads to the simultaneous formation of gold nanoparticles on the surface of the modified silica that shows both the Au-nanoparticle deposition and uniform distribution of the Au nanoparticles. Therefore, the uniformity of the Au-nanocore shell, which is reflected by the regular distribution of the Au nanoparticles on the modified silica spheres, is more effective in the case of the direct deposition of the gold nanoparticles (5–10 nm) for which the colloidal gold solution is used (Figs. 8a–f and 9a–f. In this case, the shell consists of single gold nanoparticles.

### Thermogravimetric Analysis (TGA)

**Fig. 10:**
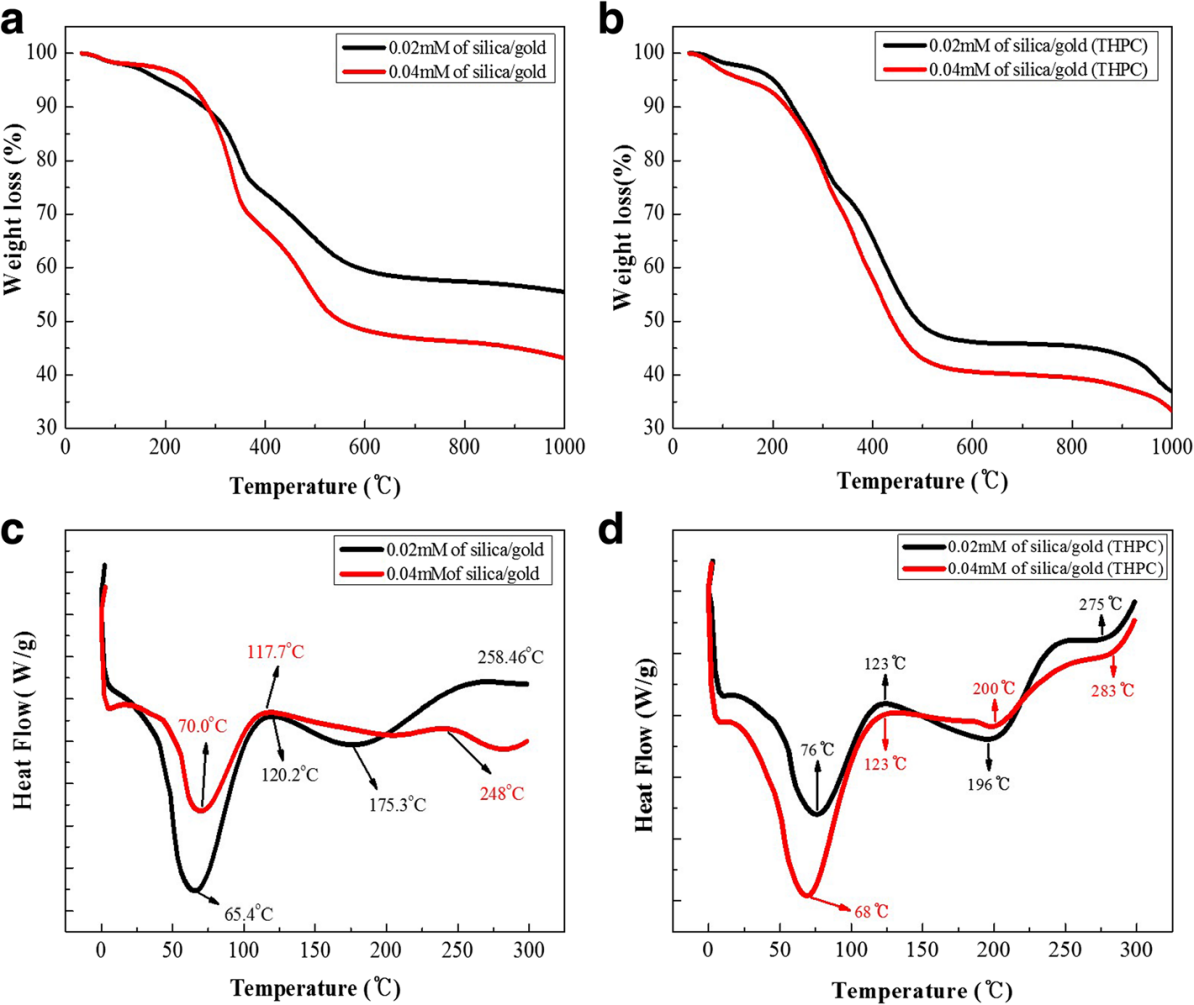
(a-b) TGA (c-d) DSC results of cellulose-amino POSS hybrid composite

The results of the TGA of the cellulose-POSS-silica/gold hybrid nanocomposites are shown in Fig. 10a, b. The thermal analysis of the hybrid-nanocomposite samples is carried out under a stream of nitrogen at a heating rate of 10 °C/min. For the TGA results, temperatures from 10 to 1000 °C are used, and the observed weight loss appears in three stages, as follows: The first stage of degradation is from 85 to 100 °C, the second stage of degradation is from 100 to 450 °C, and the third degradation is from 450 to 999 °C. The population of the silanols and water molecules (85 to 100 °C) corresponds to the water molecules that are released in the hybrid nanocomposites that are present in the outer spherical surface of the particles, as well as on the inner-pore walls. The surface of the spherical silica consists of a very small portion of free silanols, a large amount of hydrogen-bonded silanols, and adsorbed water molecules. The intensive thermal degradation of the cellulose-hybrid materials is observed between 100 and 450 °C for the cellulose-POSS hybrid-nanocomposite materials. This increase of the degradation temperature shows that the strong organic/inorganic-phase interaction greatly influences the thermal resistance. The third step of the thermal-decomposition curve indicates a correspondence to the cellulose-POSS-silica/gold with the addition of inorganic content. The third degradation shows losses from 530 to 999 °C and char residue of 44.45% at 998.5 °C. The amount of inorganic moieties that are present in the cellulose-POSS hybrid nanocomposites is therefore increased according to the thermal stability.

Figure 10b shows the thermal properties of the hybrid nanocomposites in the presence of PVA and THPC, whereby the silica/gold hybrid shows a 34.5% char residue at 999 °C. The TGA regarding both methods for the silica/gold hybrid shows a greater thermal stability compared with those of a previous report [13–16]. Differential scanning calorimetry (DSC) is one of the important thermal-characteristic properties regarding the cellulose-POSS-silica/gold hybrid composites. The DSC results are indicative of the pure cellulose and the am-POSS-grafted cellulose hybrids [15, 16]. The DSC plots for the am-POSS-grafted cellulose hybrids respectively show the first endothermic peaks at the temperatures of 80.47 and 78.29 °C. These endothermic peaks (Fig. 10c, d) are probably associated with the removal of the water from the am-POSS-grafted cellulose materials that is due to the decrease of the amino-POSS amount. The cellulose shows the second endothermic peak at 358.92 °C. The endothermic change that is shown in the DSC plot for the cellulose is associated with the decomposition processes that may occur within the chemical-heating process. The cellulose-POSS hybrid nanocomposites respectively show the second endothermic peaks at 366 and 364 °C. The thermal properties of the am-POSS-grafted cellulose are different from those of the pure-cellulose polymer because of the difference between the organic/inorganic hybrids. The endothermic peaks are estimated according to the interaction between the organic components and the inorganic components. The DSC plots of the am-POSS-bonded cellulose hybrids also indicate that the smallness of the two endothermic peaks is due to the interaction of the organic/inorganic hybrids. In addition, the cellulose-POSS-grafted silica-gold hybrid results improve the Tg, and the melting temperature increases due to an interfacial bonding between the two components [17–28].

### BET Analysis of Cellulose-POSS-Silica/Gold Hybrid Nanocomposites

**Fig. 11:**
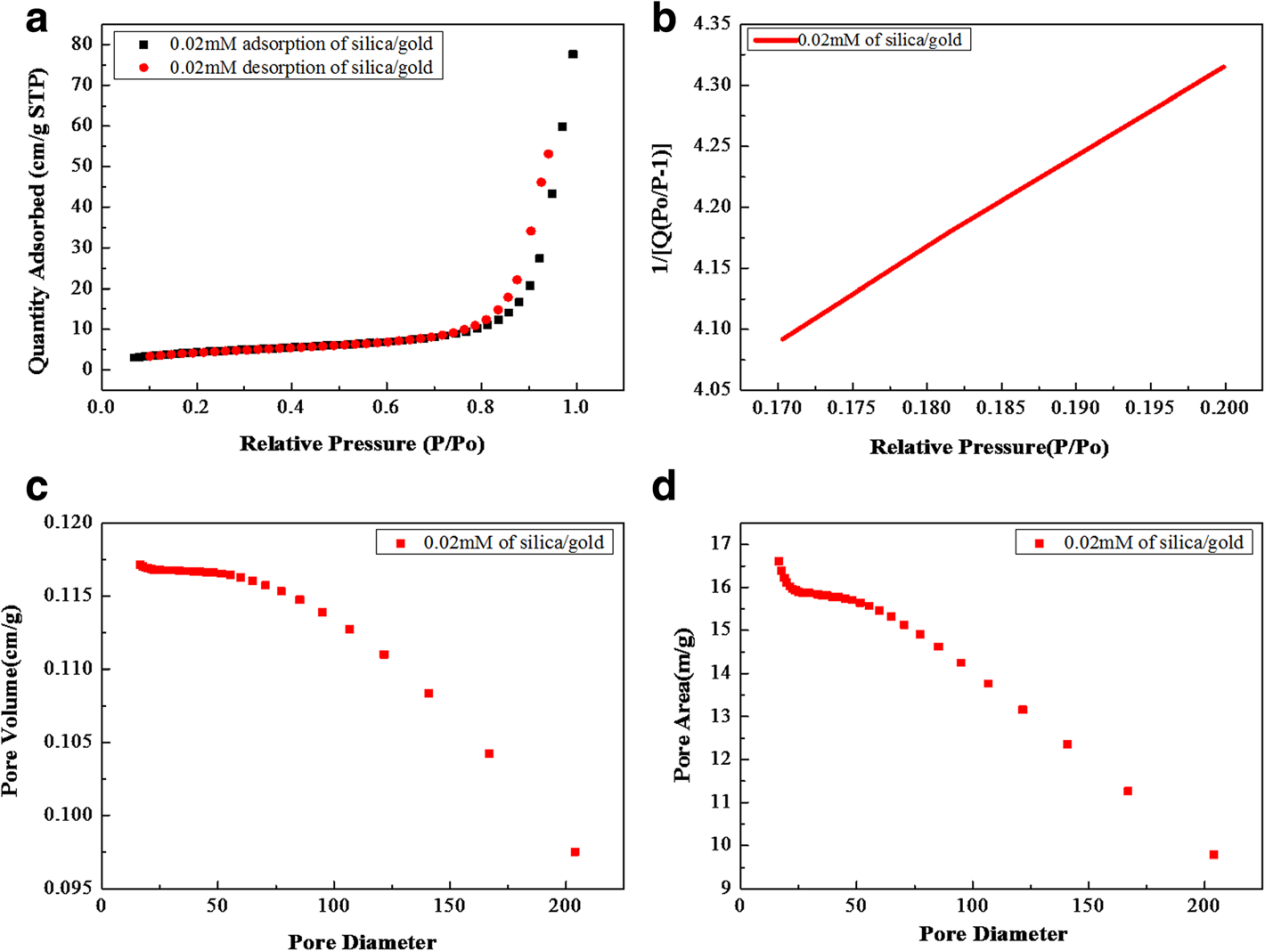
(a-d) BET results of cellulose-amino POSS hybrid composite by sol-gel process

**Fig. 12:**
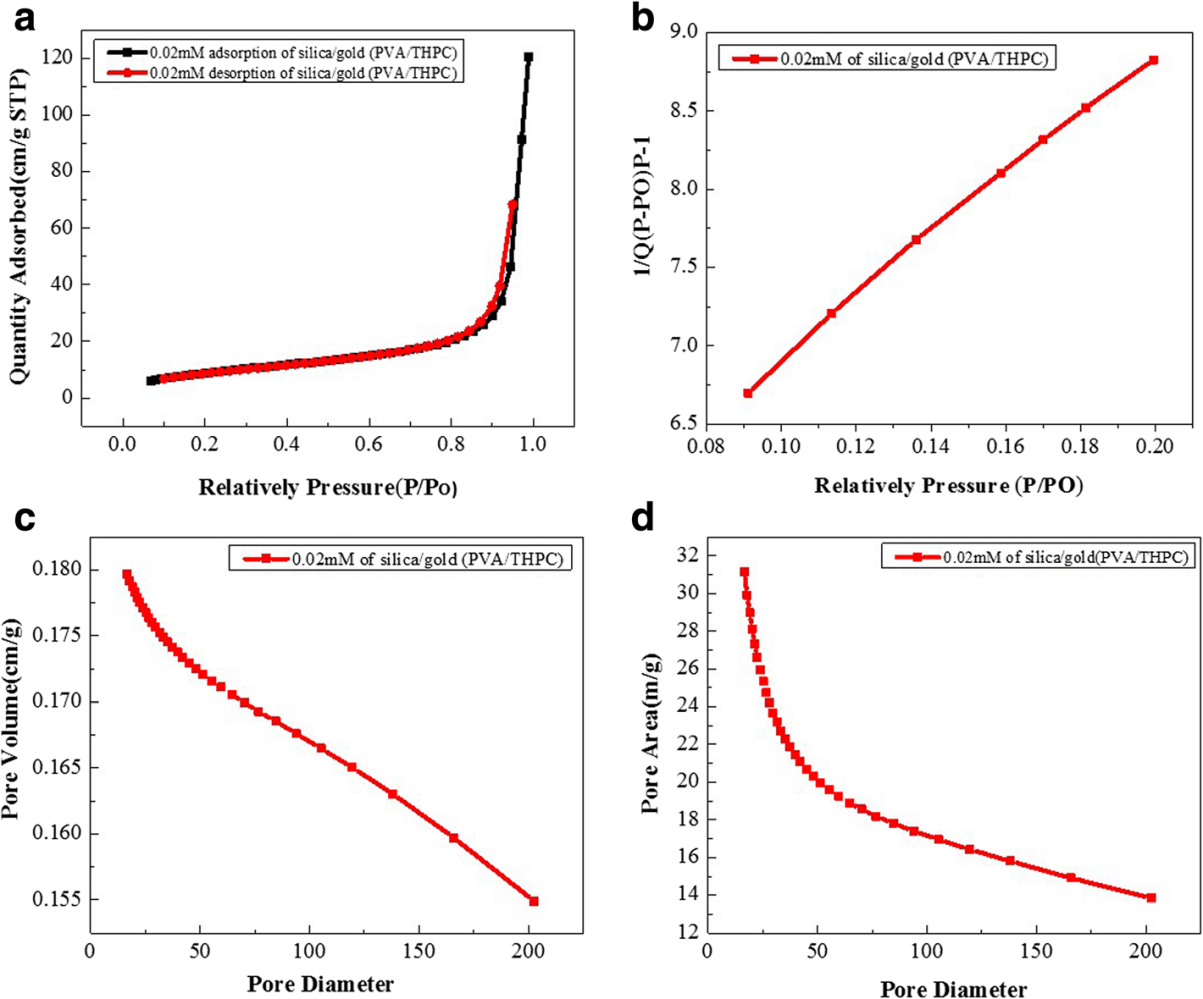
(a-d) BET results of cellulose-amino POSS hybrid composite by PVA/THPC process

The nitrogen adsorption/desorption isotherms (Fig. 11a–d) of the porous gold/silica nanocomposite samples after the calcinations results. Because of the hybrid samples, the specific surface area and the micropore volume of the cellulose-POSS-silica/gold nanocomposites are analyzed using the BET analysis, as shown in Fig. 8a–d. The results of the hybrid nanocomposites show the values of the specific surface area and the micropore volume. The BET results of the hybrid nanocomposites that are calculated using the surface analysis are as follows: single-point surface area of P/Po = 15.0295 (m/g), BET surface area = 16.644 (m/g), BJH-adsorption cumulative surface area of pores = 16.61 (m/g), BJH-desorption cumulative surface area of pores = 20.695 (m/g), adsorption of average pore width (4V/A) by BET = 288.51, BJH-adsorption average pore diameter (4V/A) = 281.99, and BJH-desorption average pore diameter (4V/A) = 231.37. In addition, the BET results of PVA/THPC process via silica/gold hybrid composite are shown in Fig. 12a–d. From these results, the single-point surface area at P/Po = 30.7536 (m/g), BET surface area = 34.1802 (m/g), BJH-adsorption cumulative surface area of pores = 31.148 (m/g), BJH-desorption cumulative surface area of pores = 35.8813 (m/g), adsorption average pore width (4V/A) = 218.04, BJH-adsorption average pore diameter (4V/A) = 230.75, and BJH-desorption average pore diameter (4V/A) = 206.33. Therefore, the comparative surface area and cumulative surface increases in the case of PVA/THPC via silica/gold hybrid composite.

## Conclusions

In this paper, cellulose-POSS-silica/gold hybrid nanocomposites are synthesized using an in situ sol-gel process in the presence of γ-APTES, PVA, and THPC. Both of the samples show the homogeneous formation of cellulose-POSS-silica/gold hybrid nanocomposites in the stable colloids. From the colloid nanoparticles, the uniform core/shell consisting of gold nanoparticles is formed on the surface of γ-aminopropyl-modified silica/gold hybrid composites. The first method uses the colloidal gold solution to form the shell on the modified silica core in the hybrid composites. The second method involves the formation and simultaneous deposition of silica/gold nanoparticles in the presence of PVA and THPC, whereby the HAuCl_4_ is reduced with formaldehyde. A comparison of both deposition methods indicates that the direct deposition of colloidal gold on the modified silica particles affords a more-uniform and homogeneous distribution of the Au nanoparticles; therefore, the deposition can be easily controlled to achieve the desired size and concentration of the gold nanoparticles on the silica surface in the presence of a coupling agent and surface modifiers. The homogeneity of the hybrid nanocomposites is influenced by the hydrolysis rate and the condensation reaction of the alkoxysilanes, which plays an important role in the sol-gel process; this might be due to the amounts of hydrochloric acid and the tetraethoxysilane/gold precursors in the presence of γ-APTES. The hybrid nanocomposites indicate that an optical transparency and a thermal stability are achieved compared to the pristine cellulose-POSS materials. The XRD results show crystalline behavior in the low-temperature PVA/THPC via silica/gold hybrid nanocomposites. The hybrid nanocomposites represent the achievement of thermal stability, PL behavior, surface morphology, and a controlled particle size via a coupling agent or surface modifiers.
