# Erratum to Nanostructured Silica/Gold-Cellulose-Bonded Amino-POSS Hybrid Composite via Sol-Gel Process and Its Properties

**DOI:** 10.1186/s11671-017-2228-0

**Published:** 2017-07-17

**Authors:** Sivalingam Ramesh, Heung Soo Kim, Young-Jun Lee, Gwang-Wook Hong, Joo-Hyung Kim

**Affiliations:** 1 Department of Mechanical, Robotics and Energy Engineering, Dongguk University-Seoul, Pil-dong, Jung-gu, Seoul, 100-715 South Korea; 2 Department of Mechanical Engineering, Inha University, Inha-ro 100, Nam-gu, Incheon, 402-751 South Korea

## Erratum

In the original publication [1] was an error with the name of author Young-Jun Lee.

Incorrect name: Young-June Lee

Correct name: Young-Jun Lee

The original article has been updated to rectify this error.
